# Protein intrinsically disordered region prediction by combining neural architecture search and multi-objective genetic algorithm

**DOI:** 10.1186/s12915-023-01672-5

**Published:** 2023-09-07

**Authors:** Yi-Jun Tang, Ke Yan, Xingyi Zhang, Ye Tian, Bin Liu

**Affiliations:** 1 School of Computer Science and Technology, Beijing Institute of Technology, Haidian District, No. 5, South Zhongguancun Street, Beijing, 100081 China; 2 School of Artificial Intelligence, Anhui University, Hefei, 230601 China; 3 Institutes of Physical Science and Information Technology, Anhui University, Hefei, 230601 China; 4 Advanced Research Institute of Multidisciplinary Science, Beijing Institute of Technology, Beijing, 100081 China

**Keywords:** Intrinsically disordered regions (IDRs), Neural architecture search (NAS), Length-dependent models

## Abstract

**Background:**

Intrinsically disordered regions (IDRs) are widely distributed in proteins and related to many important biological functions. Accurately identifying IDRs is of great significance for protein structure and function analysis. Because the long disordered regions (LDRs) and short disordered regions (SDRs) share different characteristics, the existing predictors fail to achieve better and more stable performance on datasets with different ratios between LDRs and SDRs. There are two main reasons. First, the existing predictors construct network structures based on their own experiences such as convolutional neural network (CNN) which is used to extract the feature of neighboring residues in protein, and long short-term memory (LSTM) is used to extract the long-distance dependencies feature of protein residues. But these networks cannot capture the hidden feature associated with the length-dependent between residues. Second, many algorithms based on deep learning have been proposed but the complementarity of the existing predictors is not fully explored and used.

**Results:**

In this study, the neural architecture search (NAS) algorithm was employed to automatically construct the network structures so as to capture the hidden features in protein sequences. In order to stably predict both the LDRs and SDRs, the model constructed by NAS was combined with length-dependent models for capturing the unique features of SDRs or LDRs and general models for capturing the common features between LDRs and SDRs. A new predictor called IDP-Fusion was proposed.

**Conclusions:**

Experimental results showed that IDP-Fusion can achieve more stable performance than the other existing predictors on independent test sets with different ratios between SDRs and LDRs.

**Supplementary Information:**

The online version contains supplementary material available at 10.1186/s12915-023-01672-5.

## Background

Intrinsically disordered regions (IDRs) are protein regions lacking stable three-dimensional structure [1, 2]. IDRs play essential roles in a broad range of biological functions [1, 3], such as assembler, flexible linker, and protein phosphorylation. IDRs are also correlated with several diseases [3], such as cancer and genetic diseases. Therefore, accurate identification of IDRs is an important fundamental task for studying protein functions and drug design.

With the rapid increase of the number of proteins in recent years, we need to develop faster and more effective methods to identify IDRs. With the help of machine learning algorithms, several computational models have been proposed. For example, DISOPRED [4] uses the evolutional information generated by PSI-BLAST [5] as the input of neural networks (NNs). DISOPRED3 [6] uses support vector machines (SVMs) to replace the neural networks and has improved the performance. With the development of deep learning techniques, more effective protein sequence features can be obtained, such as SPOT-Disorder [7] and AUCpreD. SPOT-Disorder proposes a model built with Bi-directional long short-term memory (Bi-LSTM) to capture the global information. AUCpreD [8] improves the predictive performance by combining convolutional neural network and conditional random fields (CRFs). Some methods combine different predictors into one model to capture the differences and commonalities of different models [2]. For example, MFDp [9] integrates three models DISOPRED2 [10], DISOclust [11] and IUCpred [12]. IDP-Seq2Seq [13] integrates three models trained by LDR dataset, SDR dataset, and mixed dataset. IDP-Seq2Seq is based on the Seq2Seq and attention mechanism to capture more comprehensive features. SPOT-Disorder2 [14] integrates five deep learning networks fusing residual convolution network and long short-term memory (LSTM).

**Fig. 1:**
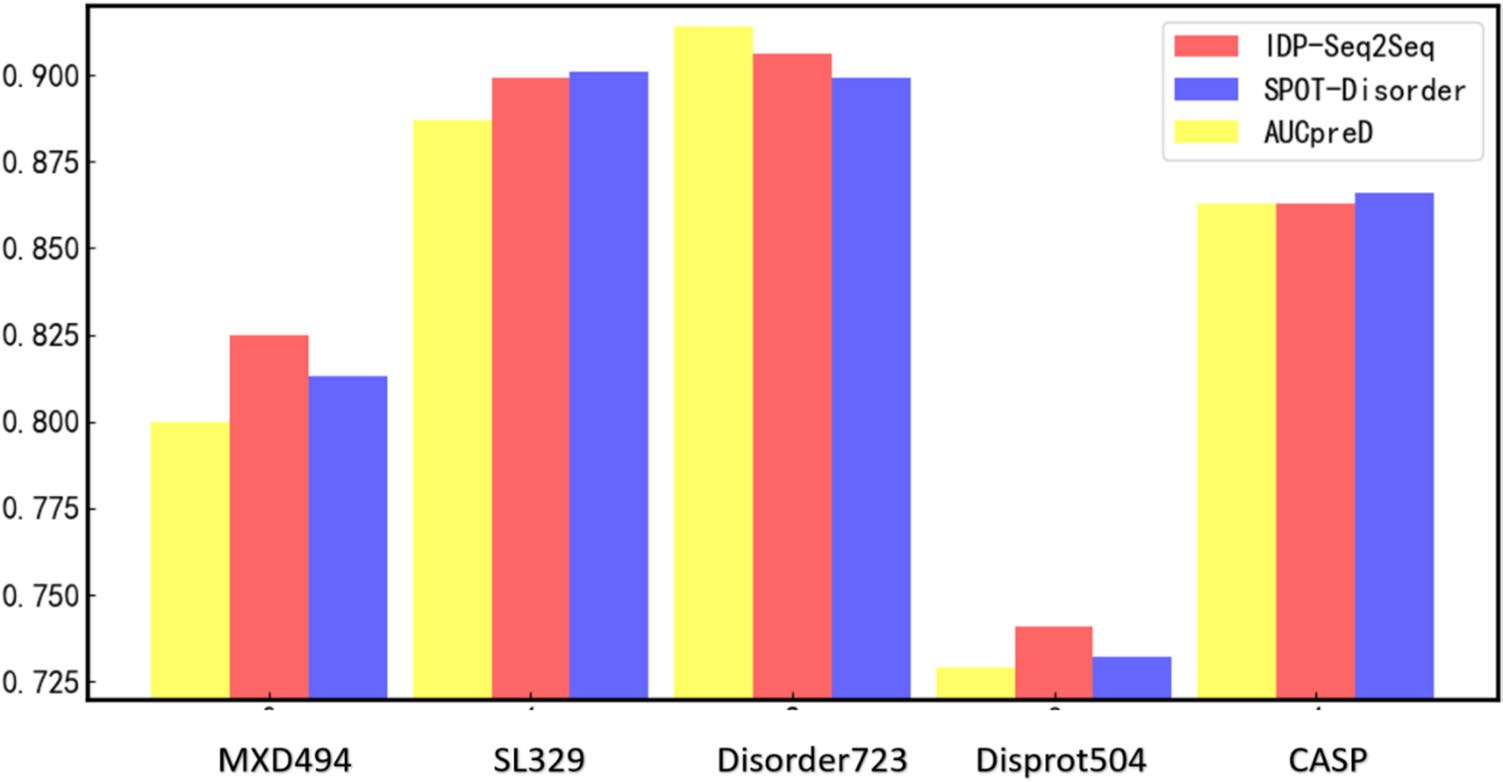
The AUC values of AUCpreD, SPOT-Disorder, and IDP-Seq2Seq on five independent test datasets with different ratios between SDRs and LDRs, including MXD494, SL329, Disorder723, Disprot504, and CASP. Detailed information about the independent test datasets is listed in Additional file 1: Table S1, and the corresponding results of different methods are shown in Additional file 1: Table S2-S6 [6–13, 15–26]

IDRs are divided into short disordered regions (SDRs) and long disordered regions (LDRs). Generally, LDRs are defined as disordered regions with more than 30 residues, while SDRs are shorter than 30 residues [15]. LDR protein is a protein sequence with at least one LDR, and SDR protein is a protein with at least one SDR but without LDR. Because SDRs and LDRs have different features [15], it is difficult for the computational methods to achieve stable performance for predicting both SDRs and LDRs. For example, we used three well-documented methods, IDP-Seq2Seq, SPOT-Disorder, and AUCpreD to test on five independent test datasets with different ratios between LDRs and SDRs, including MXD494, SL329, Disorder723, Disprot504, and CASP (see Fig. 1). SPOT-Disorder and AUCpreD are two top-performing predictors proved by a recent review by Liu et al. [2]. IDP-Seq2Seq applies the algorithm derived from natural language processing to protein disorder prediction. These three methods are typical methods that are widely used and have good performance, but the results show that the performance of these methods across different datasets is not stable. The reason is that these predictors can only accurately predict LDRs or SDRs, but they failed to accurately predict both the SDRs and LDRs. Furthermore, the existing methods ignore the fully ordered proteins widely distributed in nature. Most of these methods are evaluated on test datasets consisting of disordered proteins without or with only a few fully ordered proteins. However, in real applications, users cannot realize in advance whether a protein is a disordered protein or a fully ordered protein. The neglect of fully ordered proteins by existing predictors will prevent the real-world applications of them.

Based on the similarities between the natural languages and protein sequences, algorithms derived from the field of natural language processing (NLP) have been successfully applied to protein sequence analysis [27]. Recently, various biological language models (BLMs) have been proposed and discussed, facilitating the biological sequence analysis [28]. In this regard, we integrated five linguistic models derived from NLP to improve the prediction performance, including CAN [29], HAN [30], IDP-Seq2Seq [13], CNN-LSTM [17], and LSTM-CNN [17]. Furthermore, we also employed a neural network search (NAS) model called DARTS [31, 32] to automatically optimize the neural network architectures so as to capture the hidden information failed to be captured by the other five models. For CAN, we used SDR protein dataset for training. For HAN, we used LDR proteins for training. For IDP-Seq2Seq, CNN-LSTM, LSTM-CNN, and DARTS, we used a mixture of LDR proteins, SDR proteins, and fully ordered proteins dataset for training. These six base models were fused by a multi-objective genetic ensemble algorithm to fully consider the influence of the different ratios between SDRs and LDRs on the final performance. The proposed IDP-Fusion predictor achieved more stable performance on different test datasets with different ratios between SDRs and LDRs.

## Results and discussion

### IDP-Fusion outperforms the other competing methods on independent test datasets

In order to compare the performance of IDP-Fusion with the other competing methods, IDP-Fusion was evaluated on several independent test datasets with different ratios between SDRs and LDRs, including MXD494, SL329, DISORDER723, CASP, and DISPROT504. The performance of different methods was shown in Additional file 1: Table S2-S6 [6–13, 15–26] and Table 1. Compared with Table 1 and Additional file 1: Table S2-S6 [6–13, 15–26], we can see that IDP-Fusion outperforms all the other compared models on all the five independent test datasets. To further verify the generalization of IDP-Fusion, IDP-Fusion was also evaluated on the MSDCD dataset, which is constructed by combining all five independent test datasets (see Additional file 1: Table S1). The results of IDP-Fusion and the other compared methods are shown in Table 2, from which we can see that IDP-Fusion achieves the best performance in terms of both AUC and MCC. The reasons for the better performance of IDP-Fusion are as follows: (1) IDP-Fusion combines six base predictors. The features between these predictors are complementary. Furthermore, DARTS can capture the hidden features failed to be captured by the other models. (2) During the training process, IDP-Fusion considers the influence of different ratios between SDR proteins and LDR proteins by using the multi-objective genetic ensemble algorithm, and therefore, IDP-Fusion achieves the most stable performance on different independent test datasets.

**Table 1:** Performance of IDP-Fusion on five independent test datasets (MXD494, SL329, DISORDER723, CASP, and DISPROT504)

Independent test dataset	Sn	Sp	BACC	MCC	AUC
MXD494	0.712	0.808	0.760	0.470	0.834
SL329	0.729	0.933	0.831	0.685	0.908
Disorder723	0.625	0.962	0.793	0.539	0.917
CASP	0.594	0.960	0.777	0.537	0.893
Disprot504	0.662	0.741	0.701	0.362	0.771

**Table 2:** Performance of different methods on MSDCD independent test dataset

Predictor	Sn	Sp	BACC	MCC	AUC
IDP-Fusion	0.685	0.851	0.768	**0.494**	**0.846**
DeepIDP-2L [18]	0.705	0.835	**0.770**	0.487	0.834
RFPR-IDP [17]	0.723	0.801	0.762	0.459	0.826
IDP-Seq2Seq [13]	0.676	0.842	0.759	0.475	0.824
SPOT-Disorder [7]	0.593	0.881	0.737	0.464	0.824
AUCpreD [8]	0.538	**0.901**	0.720	0.454	0.820
SPINE-D [15]	**0.775**	0.729	0.752	0.421	0.817
DISOPRED3 [6]	0.566	0.873	0.720	0.430	0.816
IUCpred-L [12]	0.551	0.864	0.707	0.404	0.775
IUCpred-S [12]	0.493	0.884	0.689	0.386	0.774

### IDP-Fusion is insensitive with the differences among different datasets

**Fig. 2:**
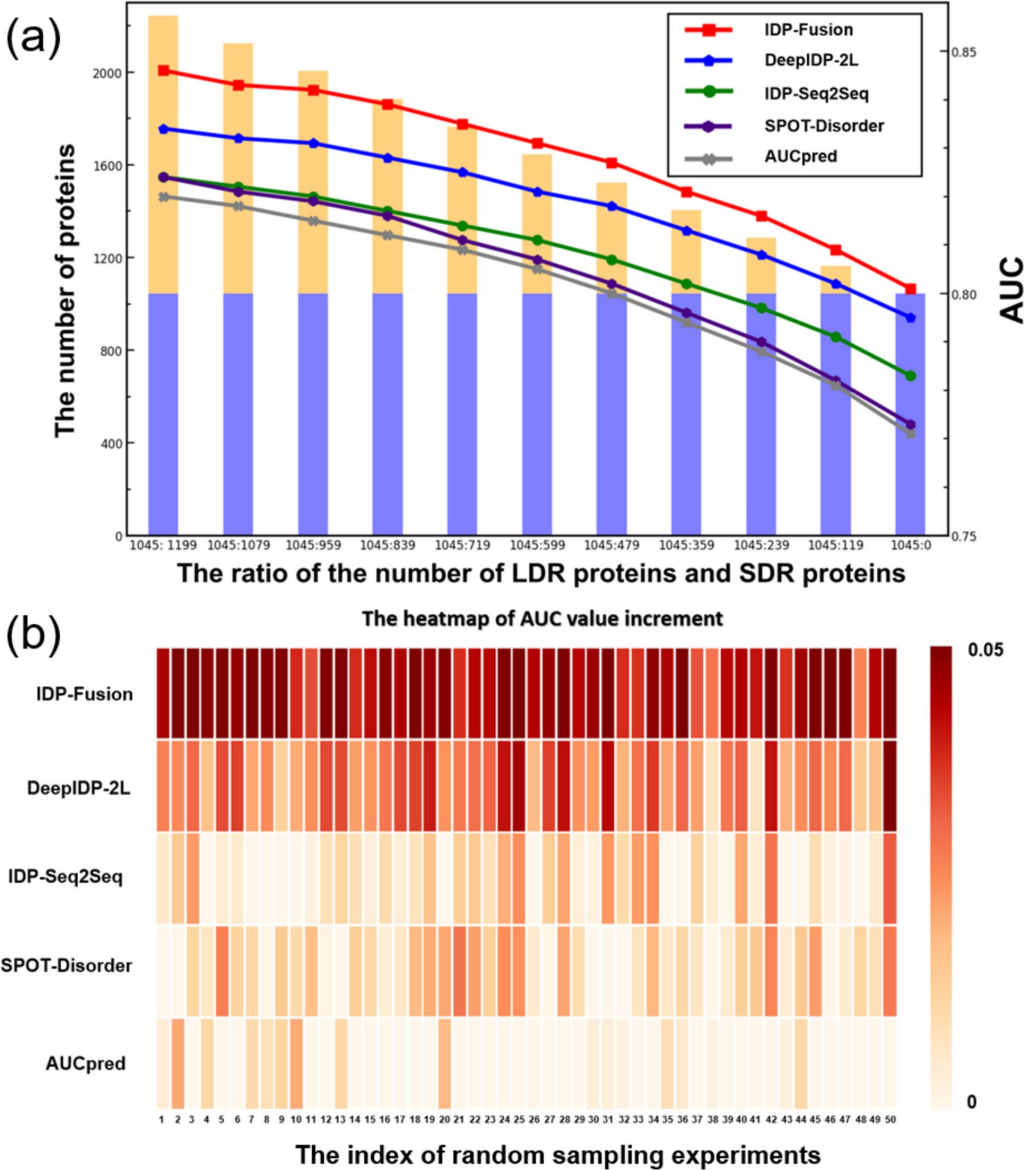
**a** The performance of IDP-Fusion, DeepIDP-2L, IDP-Seq2Seq, SPOT-Disorder, and AUCpreD evaluated on the datasets with different ratios of LDRs and SDRs. **b** The performance improvements among different methods. For each column, the values in this figure represent the performance improvement of the method labeled on the *y* axis compared with the method achieving the lowest performance in the corresponding random sampling experiment labeled in the *x* axis

From Table 1, Table 2, and Additional file 1: Table S2-S6 [6–13, 15–26], we can see that IDP-Fusion achieves stable performance on all independent test datasets. In contrast, the other predictors achieve unstable performance on different independent test datasets. For example, SPOT-Disorder achieves the best performance on the SL329 dataset, but it is only ranked as the 5-th on Disprot504. For further evaluating the performance of different predictors, we constructed eleven datasets with different ratios between SDRs and LDRs by removing SDR proteins from the MSDCD dataset. The AUC values predicted by various methods on these 11 datasets are shown in Fig. 2a. We see the following: (1) all the predictors tend to perform worse on the datasets with fewer SDRs, indicating that SDRs are easier to be predicted than LDRs; (2) the ratios between LDRs and SDRs have limited impact on the performance of IDP-Fusion, and IDP-Fusion consistently outperforms the other compared methods.

In real-world applications, for the test datasets, the ratio between LDR proteins and SDR proteins is unknown. For such a situation, a predictor with stable performance for predicting both SDR proteins and LDR proteins is preferred. In this regard, we randomly selected 400 protein sequences from MSDCD, and then these proteins were predicted by different methods. This process was repeated 50 times, and the results were shown in Fig. 2b, from which we can see that IDP-Fusion consistently outperforms the other compared methods, indicating that IDP-Fusion will be a useful method for predicting IDRs.

**Fig. 3:**
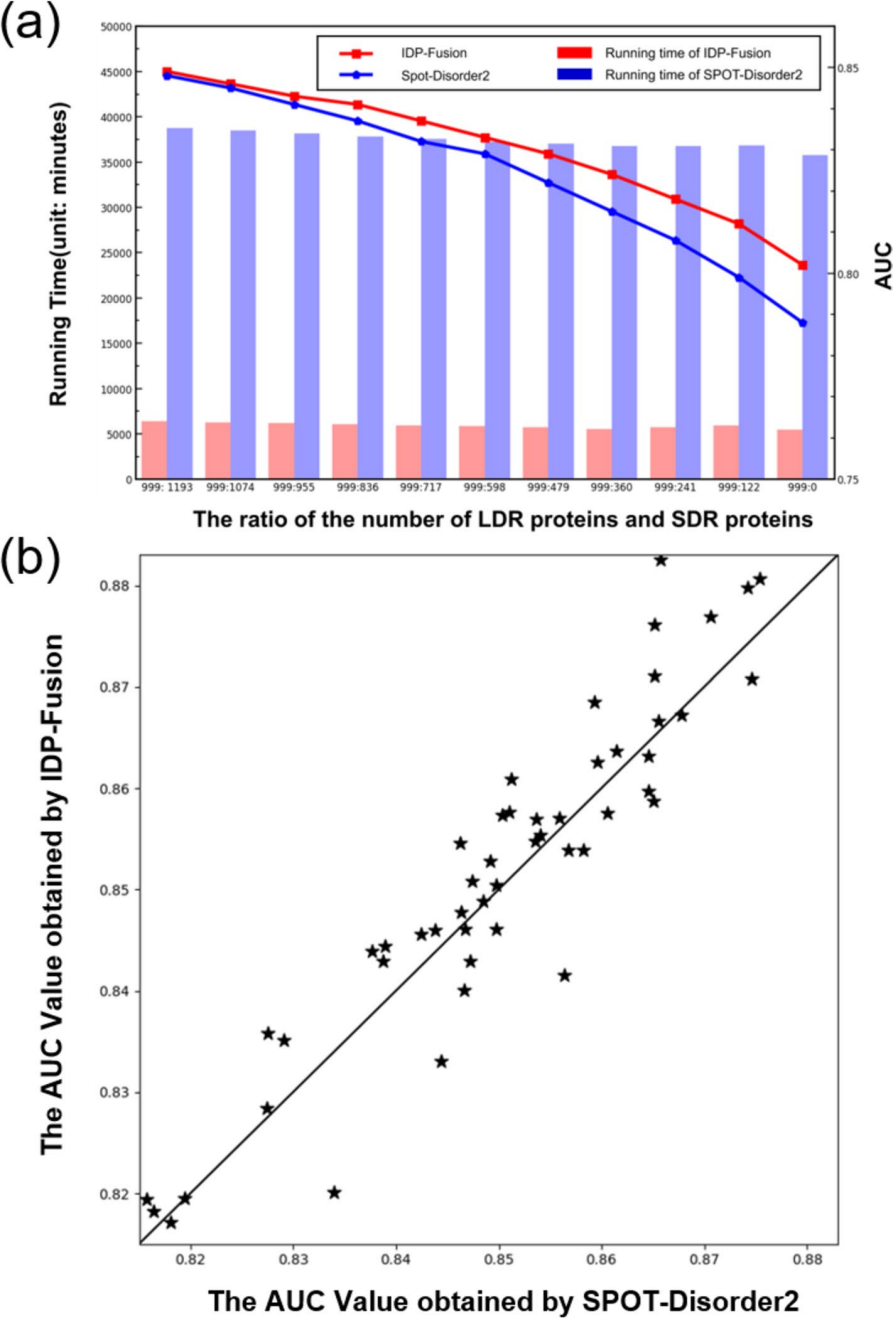
**a** The performance and computational cost comparison between IDP-Fusion and SPOT-Disorder2 on datasets with different ratios of LDR proteins and SDR proteins. **b** The 50 scatter points represent the results of the 50 random selecting experiments. If the method labeled in the *y* axis outperforms the method labeled in the *x* axis, the corresponding star point will fall on the left top part; otherwise, it will fall on the right bottom part

SPOT-Disorder2 is another efficient method for IDR prediction, achieving the state-of-the-art performance. However, SPOT-Disorder2 failed to generate results for longer proteins because of the limitation of its feature extraction methods. For fairly comparing the performance between SPOT-Disorder2 and IDP-Fusion, we removed the protein sequences which are not able be predicted by SPOT-Disorder2 from MSDCD, resulting in 999 LDR proteins and 1193 SDR proteins. These proteins were then used to evaluate the performance for both SPOT-Disorder2 and IDP-Fusion, and the results were shown in Fig. 3a, from which we can see that IDP-Fusion consistently outperforms SPOT-Disorder2, especially for the test datasets with more LDR proteins. We also compared the running time between IDP-Fusion and SPOT-Disorder2, and we found that IDP-Fusion is 10 times faster than SPOT-Disorder2 because SPOT-Disorder2 is based on 5 models with complicated features requiring more computational cost. Among the 50 randomly selected datasets based on MSDCD, IDP-Fusion outperforms SPOT-Disorder2 on most of these datasets (see Fig. 3b), further confirming that IDP-Fusion is insensitive with the different ratios between SDRs and LDRs. We conclude that IDP-Fusion is more stable with the lower computational cost for real-world applications compared with SPOT-Disoder2.

### IDP-Fusion captures commonalities and differences in protein features by fusing different models

To further explore the performance of IDP-Fusion, we evaluated the results of IDP-Fusion on the CAID1 [33]. IDP-Fusion was evaluated on two datasets from CAID1, including Disprot treating ambiguous residues in the PDB database as ordered residues and Disprot-PDB filtering out the ambiguous residues [33], and the results were shown in Table 3.

**Table 3:** Performance of different methods on Disprot and Disprot-PDB in the CAID1

Predictor	AUC^a^	Rank^b^	AUC^c^	Rank^d^	RS^e^
IDP-Fusion	0.802	2	**0.925**	1	3
DeepIDP-2L [18]	0.796	3	0.918	3	6
SPOT-Disorder2 [14]	0.76	7	0.920	2	9
fIDPnn [34]	**0.814**	1	0.873	9	10
RawMSA [35]	0.78	4	0.894	6	10
SPOT-Disorder [7]	0.757	8	0.916	4	12
AUCpreD [8]	0.757	8	0.906	5	13
ESPritz-D [36]	0.774	5	NA	NA	16
DisoMine [37]	0.765	6	NA	NA	17
Predisorder [38]	0.747	10	0.878	7	17
DISOPRED3 [6]	NA	NA	0.875	8	19
IsUnstruct [39]	NA	NA	0.868	10	21

^a^Represents the AUC value obtained by various methods on Disprot

^b^Represents the ranking of the AUC value obtained by various methods on Disprot

^c^Represents the AUC value obtained by various methods on Disprot-pdb

^d^Represents the ranking of the AUC value obtained by various methods on Disprot-pdb

^e^Represents Ranking Score (RS), which is the sum of rank^a^ and rank^b^. The smaller the value is, the better the performance of the corresponding method is. If the values of rank^a^ or rank^b^ are not available, it indicates that the corresponding method is not the top 10 best method in the CAID1. Therefore, its rank^a^ or rank^b^ is set as 11

From Table 3, we can see that IDP-Fusion achieves the most stable performance on both the Disprot and Disprot-PDB datasets in terms of RS. In contrast, the performance of the other competing methods is not stable. For example, fIDPnn is the top performing method on the Disprot, but it only ranks as the ninth best method on Disprot-PDB, indicating that this method is unstable. In contrast, IDP-Fusion obtained stable and promising results on both the two datasets. The reason is that IDP-Fusion captures commonalities and differences among protein features by fusing six different models. IDP-Fusion also participated in the CAID2. Among the 44 participating methods, IDP-Fusion is one of the top seven best performing methods on both the four datasets and achieved the most stable results [40]. Compared with CAID1, CAID2 defines four datasets and reports the F1-score index on the Disprot-noX dataset. Many methods such as fIDPnn and fIDPlr have participated in both CAID1 and CAID2. The fIDPnn predictor is the best method in CAID1, but it is the fifth best methods in CAID2; the fIDPlr predictor is the fourth best method in CAID1, but it is the eight best method in CAID2. The reason is that some more powerful methods have been proposed after CAID1, and they showed promising performance in CAID2, such as fIDPnn2 and Dispredict3.

**Fig. 4:**
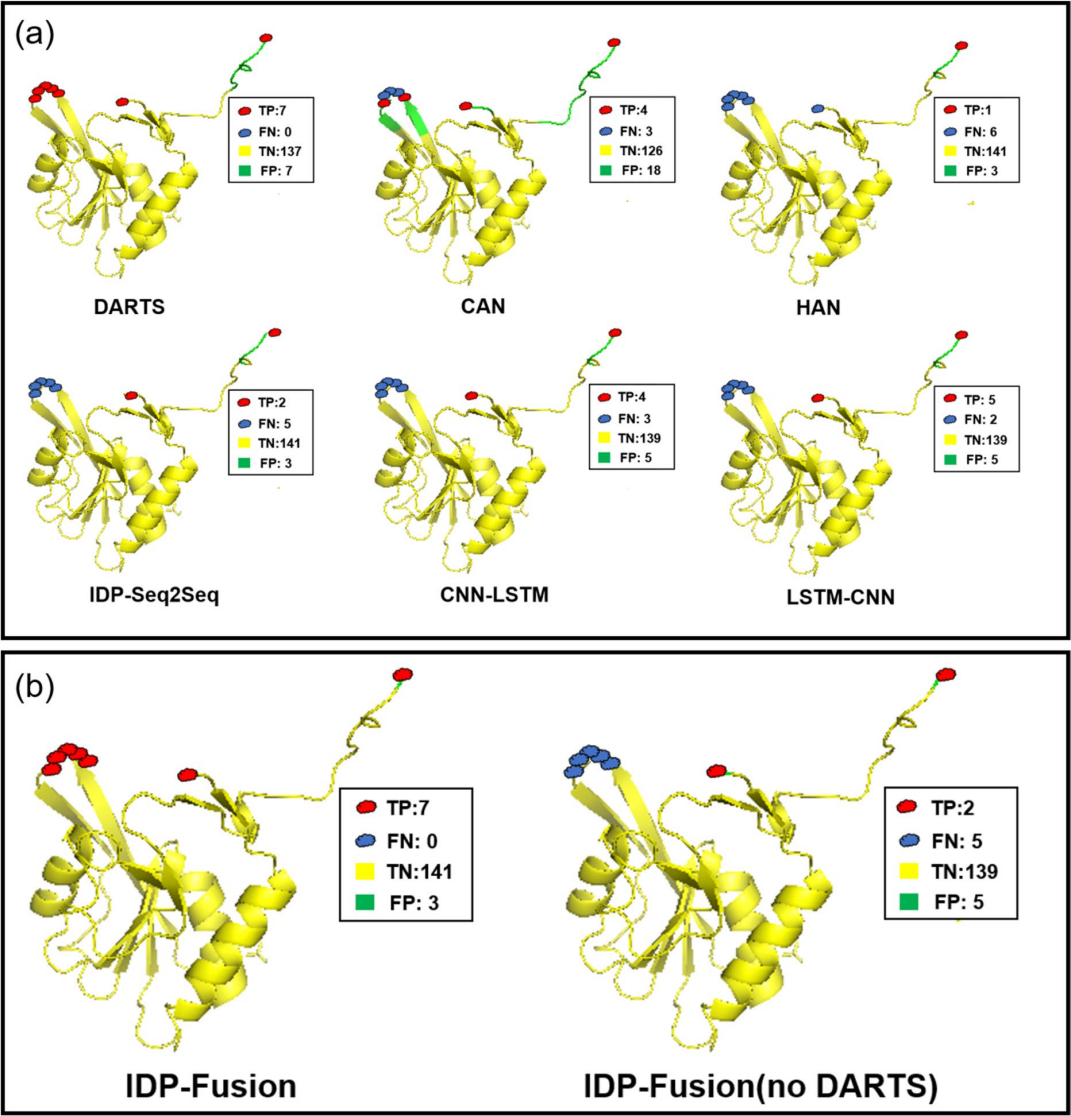
**a** The disordered residues and the other residues in protein 1a95D predicted by the six base predictors, including DARTS, CAN, HAN, IDP-Seq2Seq, CNN-LSTM, and LSTM-CNN. **b** The disordered residues and the other residues in protein 1a95D predicted by the IDP-Fusion and the IDP-Fusion without DARTS

We further explore the contribution of different base methods to the performance of IDP-Fusion. We visualized the prediction results of protein 1a95D predicted by different base methods (see Fig. 4). From Fig. 4a, we can see the following: (1) there are differences between LDRs and SDRs. For the SDR protein 1a95D, the results obtained by the method HAN trained with LDR proteins cannot identify the SDRs. It reflects that it is necessary to capture the differences between LDRs and SDRs by using the length-dependent predictors; (2) five disordered residues in the middle of protein 1a95D can be correctly predicted by DARTS, but they cannot be correctly predicted by the other methods. The reason is that DARTS is an automatically generated neural network to capture hidden features; (3) from Fig. 4b, we can see that IDP-Fusion using DARTS can correctly predict most of the SDRs, outperforming all the six base methods. These results indicate that IDP-Fusion takes the advantage of all the six base methods.

### The IDP-Fusion is more suitable for real-world application scenarios

In nature, about 20–55% proteins among all the proteins are disordered proteins [41–46]. Fully ordered proteins without IDRs are widespread in nature, but they are often ignored by the existing IDP predictors. As a result, the existing predictors tend to predict the fully ordered proteins as IDPs [17]. However, for newly sequenced proteins, the ratios between disordered proteins and fully ordered proteins are often unknown. Therefore, a predictor that can accurately predict both the IDR proteins and fully ordered proteins is highly desired. In this regard, we incorporated the fully ordered proteins into the training dataset and conducted the following experiments to verify the effectiveness of IDP-Fusion for real-world application scenarios.

**Fig. 5:**
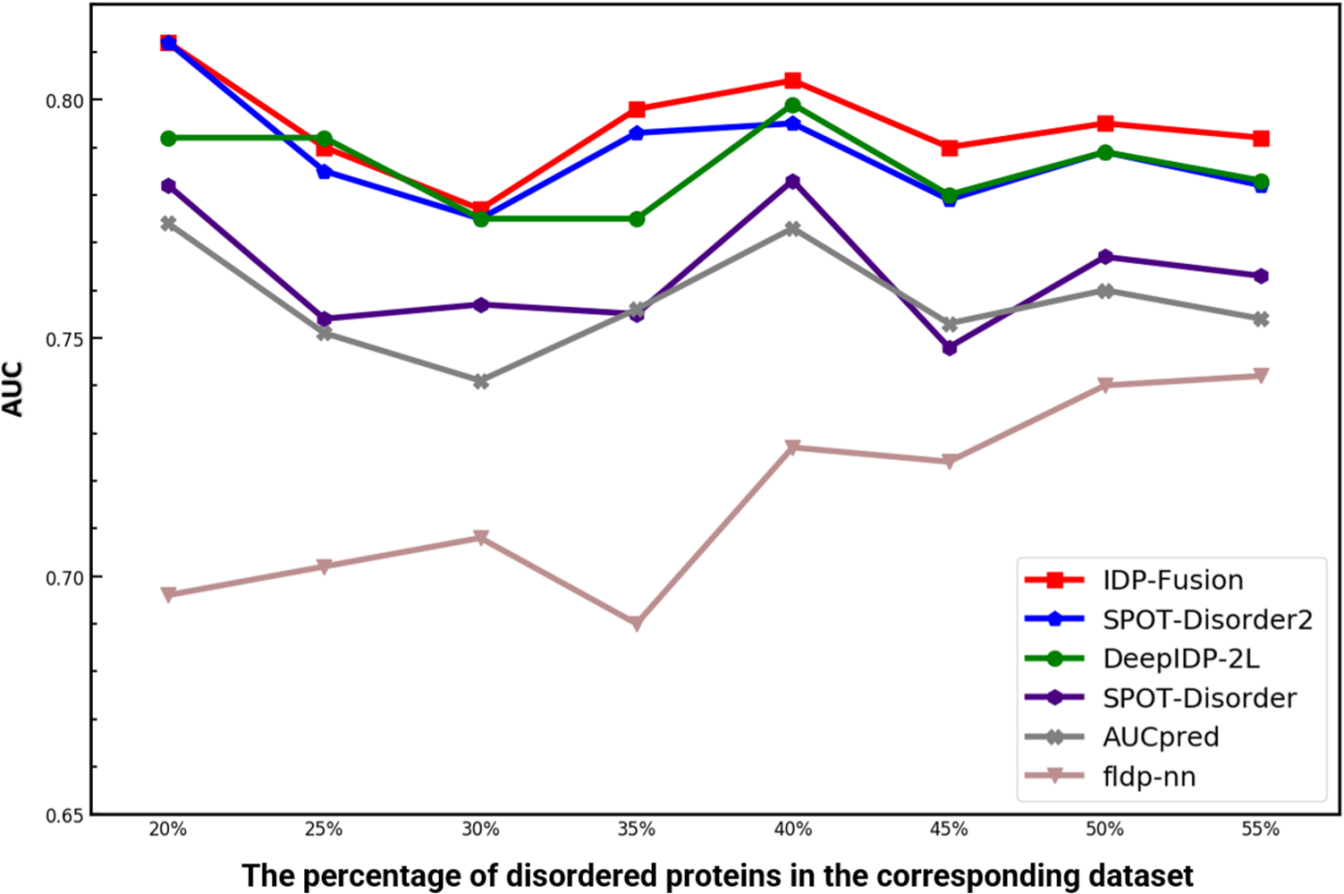
The performance of IDP-Fusion, DeepIDP-2L, IDP-Seq2Seq, SPOT-Disorder, AUCpreD, and fIDPnn evaluated on the datasets with different ratios of disordered proteins and fully ordered proteins

Eight datasets with different ratios between disordered proteins and fully ordered proteins were constructed. The statistical information of these eight datasets is listed in Additional file 1: Table S9. Five top performing methods on CAID1 were selected to compare IDP-Fusion, including fIDPnn, SPOT-Disorder2, SPOT-Disorder, AUCpreD, and DeepIDP-2L, and the results were shown in Fig. 5, from which we can see that IDP-Fusin achieved the best results on each dataset, and are more stable than the other methods.

## Conclusions

The performance of the same method varies greatly among different datasets, and various methods are ranked differently on different datasets. In order to solve this problem, we proposed a new predictor based on deep learning called IDP-Fusion. Compared with the other methods for predicting IDRs, it has the following advantages: (1) the neural architecture search employed by IDP-Fusion can capture the hidden information of the protein sequences, overcoming the disadvantages of the manually designed models only capturing the experience features; (2) the multi-objective genetic ensemble algorithm fully considers the influence of the different ratios between SDRs and LDRs on the final performance, improving the stability of IDP-Fusion; (3) we incorporate the fully ordered proteins into the training dataset to accurately predict both the IDPs and the full ordered proteins.

## Methods

### Benchmark dataset

The training dataset included 614 LDR proteins, 3024 SDR proteins, and 616 fully ordered proteins, which is constructed based on ${\mathbb{S}}_{all}^{Train}$ derived from [18] and [17] (https://disprot.org/, https://www.mobidb.org/). We removed protein sequences in ${\mathbb{S}}_{all}^{Train}$ sharing more than 25% similarities with any protein in the seven independent test datasets (see Additional file 1: Table S1) so as to avoid overestimating the performance of a predictor. We also constructed five validation datasets with different ratios between SDR proteins and LDR proteins by randomly selecting protein sequences from ${\mathbb{S}}_{all}^{Validation}$. The statistical information of these validation datasets is shown in Additional file 1: Table S7. The ${\mathbb{S}}_{all}^{Train}$ and ${\mathbb{S}}_{all}^{Validation}$ can be formulated as and the statistical information of ${\mathbb{S}}_{all}^{Train}$ and ${\mathbb{S}}_{all}^{Validation}$ are in Additional file 1: Table S8:

1 $$\left\{\begin{array}{c}{\mathbb{S}}_{all}^{Train}={\mathbb{S}}_{long}^{Train}\cup {\mathbb{S}}_{short}^{Train}\cup {\mathbb{S}}_{ordered}^{Train}\\ {\mathbb{S}}_{all}^{Validation}={\mathbb{S}}_{long}^{Validation}\cup {\mathbb{S}}_{short}^{Validation}\end{array}\right.$$

### Independent test datasets

In this study, five commonly used datasets with different ratios between SDRs and LDRs were used to evaluate the performance of different methods., including MXD494 [47], SL329 [48], DISORDER723 [19], CASP [8], and Disprot504(https://disprot.org/) [18]. To further test the generalization of various methods, the MSDCD independent test dataset was constructed by combining these five datasets. IDP-Fusion was also evaluated on the CAID1 [33]. The statistical information of the seven datasets is listed in Additional file 1: Table S1. The benchmark dataset and independent test datasets can be accessed at http://bliulab.net/IDP-Fusion/benchmark/.

### Residue representation

Three types of features were combined into IDP-Fusion, including residue-profile features, evolutionary features, and structural features [8]. Residue-profile features included seven commonly used amino acid physic-chemical properties [49]. Evolutionary-level features included position-specific frequency matrix (PSFM) and position-specific scoring matrix (PSSM) [50]. We used PSI-BLAST [5] to obtain PSSM and PSFM by searching against the nrdb90 database [51] with an *E*-value of 0.001. Besides, the evolutionary-level features included the hidden Markov model (HMM) profile generated by searching against the uniprot20_2016_02 database using HHblits software [52]. The PSSM, PSFM, and HMM features are 20-dimensional features. Structural-level features included 8-dimensional secondary structure (SS), 2-dimensional CN, and 4-dimensional HSE predicted by using SPIDER2 software tool [53], 21-dimensonal predicted residue-residue contacts (CCMs) predicted by using CCMpred software tool [54], and 1-dimensional solvent accessibility (SA) predicted by using the Sable Version 2 software tool [55]. The ablation experiments were employed to optimize the input features of different base methods, and the detailed features used in the six base methods are listed in Table 4. Because the two-dimensional convolution operation Differentiable Architecture Search DARTS [31, 32] was used in the NAS model, the corresponding input should be a three-dimensional feature matrix. A protein sequence is represented as:

2 $$\mathbf{P}= {\mathrm{R}}_{1}, {\mathrm{R}}_{2},\dots , {\mathrm{R}}_{L}$$

where ${\mathrm{R}}_{i}$ represents the $i$ th residue and $L$ is the length of **P**. The PSSM, PSFM, and HMM features of ${\mathrm{R}}_{i}$ can be represented as:

**Table 4:** The residue representation information of different base methods

Type of data	Features	Dimension
HAN	PSSM, PSFM, HHM, SS, SEVEN, SA, CCM	97
CAN	PSSM, PSFM, HHM, SS, CN, HSE, SEVEN, SA, CCM	103
IDP-Seq2Seq	PSSM, PSFM, HHM, SS, CN, HSE, SEVEN, SA, CCM	103
CNN-LSTM	PSSM, PSFM, HHM, SS, CN, HSE, SEVEN, SA, CCM	103
LSTM-CNN	PSSM, PSFM, HHM, SS, CN, HSE, SEVEN, SA, CCM	103
DARTS	PSSM, PSFM, HHM	20 × 20 × 3

3 $${\mathrm{PSSM}}_{{R}_{i}}=[{S}_{{R}_{i}}^{1},{S}_{{R}_{i}}^{2}\dots {S}_{{R}_{i}}^{20}]$$

4 $${\mathrm{PSFM}}_{{R}_{i}}=[{F}_{{R}_{i}}^{1},{F}_{{R}_{i}}^{2}\dots {F}_{{R}_{i}}^{20}]$$

5 $${\mathrm{HMM}}_{{R}_{i}}=[{H}_{{R}_{i}}^{1},{H}_{{R}_{i}}^{2}\dots {H}_{{R}_{i}}^{20}]$$

To generate the features of ${\mathrm{R}}_{i}$ fed into the DARTS, we used a sliding window with a size of 20 residues to extract the two-dimensional feature vector, and ${\mathrm{R}}_{i}$ is the 10th residue in the sliding window. The corresponding two-dimensional feature metrics of PSSM, PSFM, and HMM features of ${\mathrm{R}}_{i}$ can be represented as:

6 $${\mathrm{PSSM}}_{{R}_{i}}^{\prime}=\left[\begin{array}{ccc}{S}_{{R}_{i-9}}^{1}& \cdots & {S}_{{R}_{i-9}}^{20}\\ \vdots & \ddots & \vdots \\ {S}_{{R}_{i+10}}^{1}& \cdots & {S}_{{R}_{i+10}}^{20}\end{array}\right]$$

7 $${\mathrm{PSFM}}_{{R}_{i}}^{\prime}=\left[\begin{array}{ccc}{F}_{{R}_{i-9}}^{1}& \cdots & {F}_{{R}_{i-9}}^{20}\\ \vdots & \ddots & \vdots \\ {F}_{{R}_{i+10}}^{1}& \cdots & {F}_{{R}_{i+10}}^{20}\end{array}\right]$$

8 $${\mathrm{HMM}}_{{R}_{i}}^{\prime}=\left[\begin{array}{ccc}{H}_{{R}_{i-9}}^{1}& \cdots & {H}_{{R}_{i-9}}^{20}\\ \vdots & \ddots & \vdots \\ {H}_{{R}_{i+10}}^{1}& \cdots & {H}_{{R}_{i+10}}^{20}\end{array}\right]$$

The ${\mathrm{PSSM}}_{{R}_{i}}^{\prime}$, ${\mathrm{PSFM}}_{{R}_{i}}^{\prime}$, and ${\mathrm{HMM}}_{{R}_{i}}^{\prime}$ were treated as the three channels of R_*i*_, and they were combined leading to a three-dimensional feature matrix with a dimension of 20 × 20 × 3 (see Eq. 9), and then it was fed into DARTS.

9 $${\mathrm{Feature}}_{{R}_{i}}=[{\mathrm{PSSM}}_{{R}_{i}}^{\prime}, {\mathrm{PSFM}}_{{R}_{i}}^{\prime}, {\mathrm{HMM}}_{{R}_{i}}^{\prime}]$$

CCM represents the coevolution between residues assigning the contact probability for each residue-residue pair. The CCM of $\mathbf{P}$ can be represented as [13].

10 $${\mathbf{C}}_{\mathrm{CCM}}=\left[\begin{array}{ccc}{C}_{\mathrm{1,1}}& \cdots & {C}_{1,L}\\ \vdots & \ddots & \vdots \\ {C}_{L,1}& \cdots & {C}_{L,L}\end{array}\right]$$

We adopted a sliding window strategy to extract the CCM feature of each residue. The window size *k* was set as 21, and the CCM feature for residue *R*_*i*_ can be represented as:

11 $${\mathrm{Feature}}_{{\mathbf{R}}_{{\varvec{i}}}^{\mathbf{C}\mathbf{C}\mathbf{M}}}=\left[{C}_{i,i-\frac{k-1}{2}},{C}_{i,i-\frac{k-1}{2}+1},{\dots ,C}_{i,i}\dots ,{C}_{i,i+\frac{k-1}{2}}\right]$$

The CCM feature of each residue was finally transformed into a 21-dimensional feature vector containing local information. The missing values were set 0. The recurrent neural network was then performed on CCM features to capture the spatial information of the predicted protein structures.

### Architecture of IDP-Fusion

**Fig. 6:**
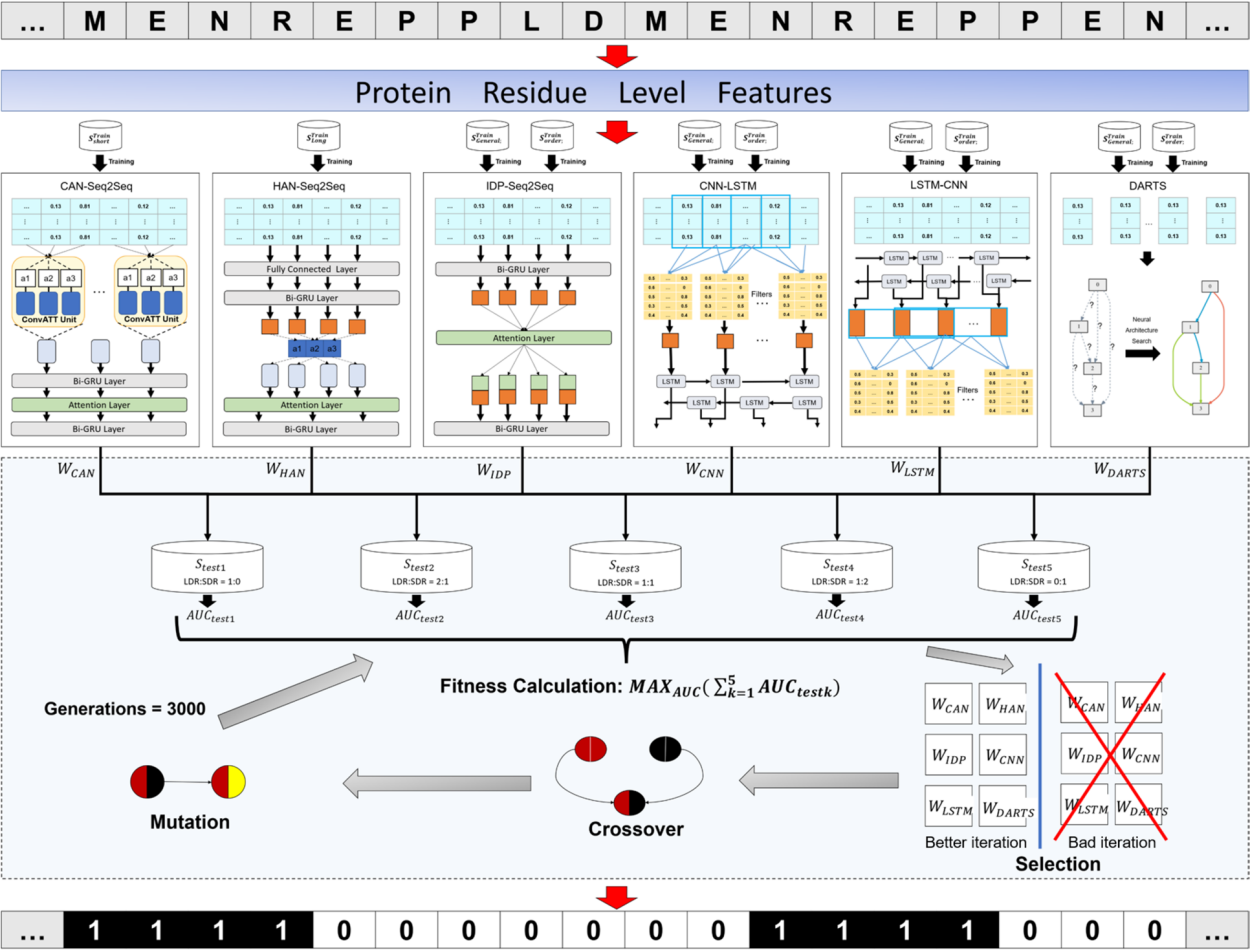
The overall architecture of IDP-Fusion. IDP-Fusion incorporated six base methods to capture complementary features of IDRs and used a multi-objective genetic algorithm to fuse the prediction probabilities of the six base methods to obtain the final prediction results (this figure can be download at the following link: http://bliulab.net/file/IDP-fusion.tif.)

The architecture of IDP-Fusion is shown in Fig. 6.

#### Five base methods derived from NLP to extract features of IDRs

IDP-Fusion fused six base methods to stably predict both the SDRs and LDRs (see Fig. 6). Among the six base methods, five models were derived from the field of natural language processing, including CAN [29], HAN [30], IDP-Seq2Seq [13], CNN-LSTM [17], and LSTM-CNN [14, 17]. CAN used the convolutional attention network to obtain the discrete distribution patterns of SDRs in protein sequences. HAN employed the hierarchical attention model to capture the sequence location of LDRs mainly located in the N’ and C’ of the sequences. IDP-Seq2Seq combined Seq2Seq and attention mechanism to capture the global and non-local correlation features of residues in IDRs. Convolutional neural networks (CNN) was used to extract local features of IDRs, and long short-term memory (LSTM) was used to extract global features of IDRs. The CNN and LSTM were combined to obtain both the local features and global features of IDRs. Two models CNN-LSTM and LSTM-CNN were constructed inspired by SPOT-Disorder2 [14].

#### The neural architecture search network

**Fig. 7:**
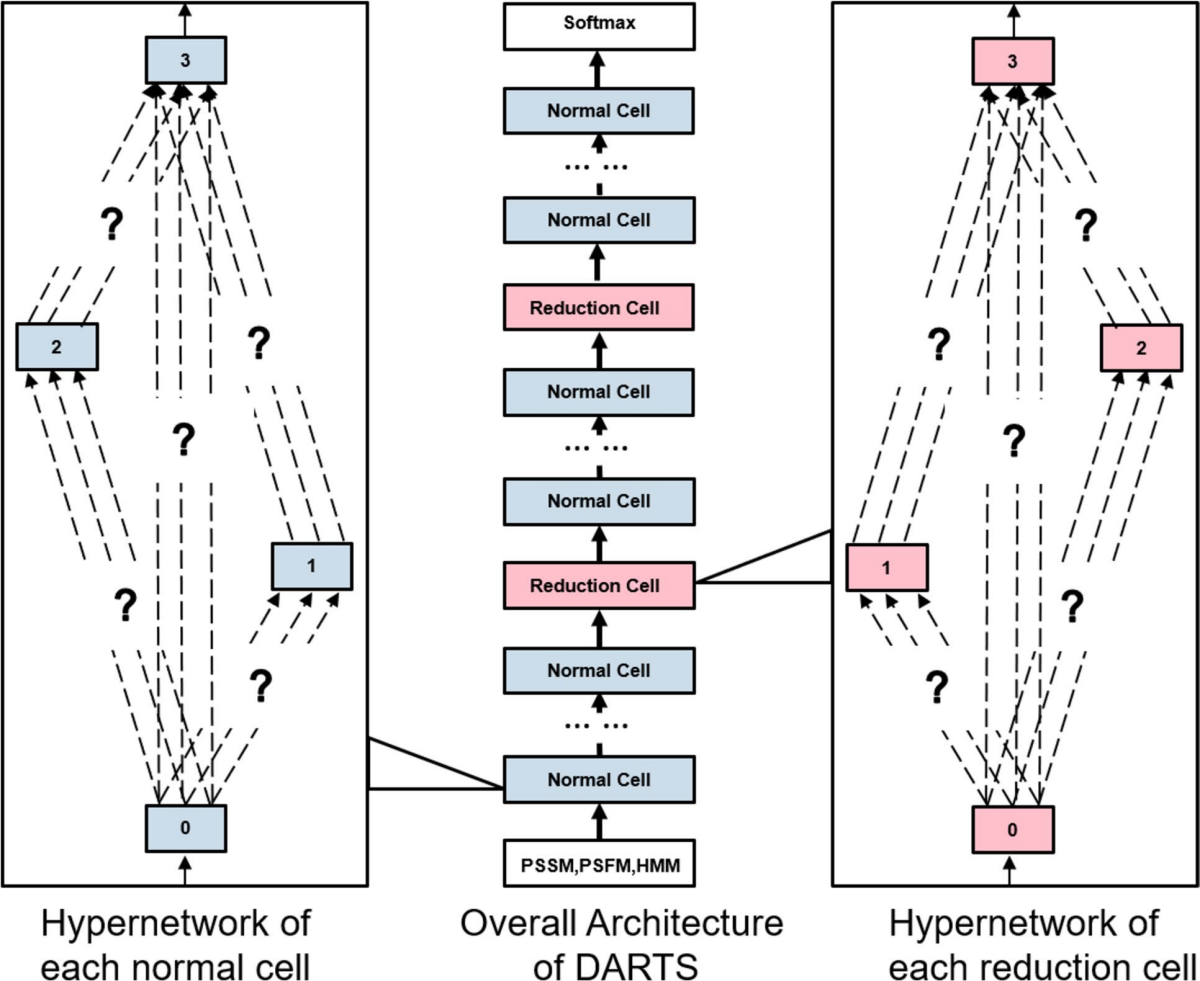
The structure of the DARTS model employed by IDP-Fusion

The aforementioned base models can capture various features of IDRs and achieve complementary prediction results. All these five base methods are based on the deep neural networks manually designed by experience. However, the unknown features or hidden features are important for IDR prediction as well. Furthermore, they are even complementary with the features extracted by the five base methods. In this regard, we employed the neural architecture search (NAS) model DARTS [31, 32] to capture the hidden features of protein sequences. The DARTS algorithm automatically constructs the optimal architecture for the normal cell and reduction cell in convolutional neural networks (see Fig. 7). DARTS constructs the structure $a$ by minimizing the loss function on ${\mathbb{S}}_{all}^{Validation}$(see Eq. 12) [32] and optimizes the corresponding parameters ${w}^{*}\left(a\right)$ by iterating the structure $a$ on the ${\mathbb{S}}_{all}^{train}$ (see Eq. 13) [32].

12 $$a={\mathrm{min}}_{a} {\mathrm{Loss}}_{val}({w}^{*}\left(a\right), a)$$

13 $${w}^{*}\left(a\right)={\mathrm{argmin}}_{w}{\mathrm{Loss}}_{train}\left(w,a\right)$$

The different nodes in the normal cell and reduction cell shown in Fig. 7 represent the feature vectors of different stages. The feature node of each stage is connected to the feature nodes of all its predecessor stage through the operation $o$ [32].

14 $$x_j={\textstyle\sum_{i<j}}o^{(i,j)}(x_i)$$

where $x_j$  represents the *j*th feature node, and ${x}_{i}$ represents the predecessor node of ${x}_{j}$. The goal of DARTS is to obtain the specific operation ${o}^{(i,j)}$ from all the optional operation spaces $\mathrm{\rm O}$. The optional operation spaces $\mathrm{\rm O}$ are a collection of a series of discrete operations, including convolution, pooling, residual convolution, and the other operations. In order to make the search space continuous, we assign a weight $\alpha$ to each operation. In this way, the search task is simplified to learn the weight $\alpha$,

15 $${\overline{o} }^{\left(i,j\right)}\left(x\right)= {\sum}_{o\in O}\frac{\mathrm{exp}\left({{\alpha }_{o}}^{\left(i,j\right)}\right)}{{\sum }_{{o}{\prime}\in O}\mathrm{exp}\left({\alpha }_{{o}{\prime}}^{(i,j}\right)}o(x)$$

After the search is completed, the operation with the largest weight is selected as the specific operation between ${x}_{i}$ and ${x}_{j}$. As a result, the discrete structure is obtained again. Finally, the specific operations between all ${x}_{i}$ and ${x}_{j}$ are obtained, thereby determining a structure $a$, and then optimizing the parameters $w$ of the structure $a$. DARTS obtains the best structure by continuously and automatically iterating in the process of learning feature. Because the optimal model is automatically selected during the feature optimization process, the model obtained by DARTS can capture the hidden information, which cannot be captured by the other five base models.

#### Multi-objective genetic ensemble algorithm

The prediction probabilities generated by the six complementary base methods should be fused to make the final prediction. The currently fusion strategy ignoring the stability of the model on independent test datasets with different ratios between LDRs and SDRs, such as the average fusion strategy [14]. In order to make the IDP-Fusion predictor insensitive with the different ratios between SDRs and LDRs, we introduced a fusion approach called multi-objective genetic algorithm (MOGA) to automatically optimize the weights of the six base methods. Five validation datasets with different ratios between SDRs and LDRs were constructed (see Additional file 1: Table S7), and the weights were optimized by maximizing the sum of the AUC scores of the six base predictors on these validation datasets (see Eq. 16) instead of the AUC of a certain dataset. We used genetic algorithm [56] to optimize the sum of the multi-datasets to obtain the weights of different base methods. For a residue *r*, its prediction probability is the sum of the weighted probabilities of the six base methods.:

16 $$\mathrm{Fitness\;calculation}={\mathrm{MAX}}_{AUC}( {\sum }_{k=1}^{5}{AUC}_{testk})$$

Compared with the average fusion strategy, the performance of MOGA is better and more stable on independent test datasets with different ratios between LDRs and SDRs (see Additional file 1: Table S15).

### Performance evaluation

The evaluation indicators used in this study are as follows:

17 $$\left\{\begin{array}{c}Sn=\frac{TP}{TP+FN}\\ Sp=\frac{TN}{TN+FP} \\ BACC=\frac{1}{2}\left(\frac{TP}{TP+FN}+\frac{TN}{TN+FP}\right) \\ MCC=\frac{\left(TP\times TN\right)-\left(FP\times FN\right)}{\sqrt{\begin{array}{c}\left(TP+FP\right)\left(TP+FN\right)\left(TN+FP\right)\left(TN+FN\right)\end{array}}}\\ AUC: the\;area\;under\;the\;ROC\; curve\end{array}\right.$$

where *TP*, *FP*, *TN*, and *FN* represent the number of true positives, false positives, true negatives, and false negatives, respectively.

### Supplementary Information

**Additional file 1: Supplementary Tables.** The statistical information of six independent test datasets is listed in Table S1. Performance of various methods on MXD494, SL329, DISORDER723, Disprot504 and CASP is listed in Table S2-S6 [6–13, 15–26]. The statistical information of five validation datasets is listed in Table S7. The statistical information of training and validation datasets is listed in Table S8. The statistical information of eight test datasets simulating real-world application scenarios is listed in Table S9. The performance of different combining feature of five base methods in Table S10-14. The performance comparison of multi-objective genetic algorithm and averaging algorithm on MSDCD dataset in Table S15.

## Data Availability

The IDP-Fusion webserver is accessible at http://bliulab.net/IDP-Fusion/benchmark/, and all data utilized in this study is available at http://bliulab.net/IDP-Fusion/benchmark/. The code and datasets used in this study can be found in online repositories. The name of the repository and accession number for the data reported in this paper is zenodo, 10.5281/zenodo.8190096 [57]. All data generated or analyzed during this study are included in this published article, its supplementary information files, and publicly available repositories.

## Abbreviations

IDRs: Intrinsically disordered regions
LDRs: Long disordered regions
CNN: Convolutional neural network
LSTM: Long short-tern memory
NAS: Neural architecture search
NNs: Neural networks
SVMs: Support vector machines
Bi-LSTM: Bi-directional long short-term memory
CRFs: Conditional random fields
NLP: Natural language processing
BLMs: Biological language models
PSSM: Position-specific scoring matrix
PSFM: Position-specific frequency matrix
HMM: Hidden Markov model
CCMs: Residue-residue contacts
MOGA: Multi-objective genetic algorithm
