# Supplementary material for: Protein intrinsically disordered region prediction by combining neural architecture search and multi-objective genetic algorithm
Source: BMC Biol. 2023 Sep 7;21:188. doi: 10.1186/s12915-023-01672-5 (PMC10483879; doi:10.1186/s12915-023-01672-5)
Supplement: Supplementary file 1 — Additional file 1: Supplementary Tables. The statistical information of six independent test datasets is listed in Table S1. Performance of various methods on MXD494, SL329, DISORDER723, Disprot504 and CASP is listed in Table S2-S6 [6–13, 15–26]. The statistical information of five validation datasets is listed in Table S7. The statistical information of training and validation datasets is listed in Table S8. The statistical information of eight test datasets simulating real-world application scenarios is listed in Table S9. The performance of different combining feature of five base methods in Table S10-14. The performance comparison of multi-objective genetic algorithm and averaging algorithm on MSDCD dataset in Table S15. [file 12915_2023_1672_MOESM1_ESM.docx]

**Additional File 1.**

The statistical information of six independent test datasets is listed in **Table S1**.

Performance of various methods on MXD494, SL329, DISORDER723, Disprot504 and CASP is listed in **Table S2-S6**.

The statistical information of five validation datasets is listed in **Table S7**.

The statistical information of training and validation datasets is listed in **Table S8**.

The statistical information of eight test datasets simulating real-world application scenarios is listed in **Table S9**.

The performance of different combining feature of five base methods in **Table S10-14**.

The performance comparison of multi-objective genetic algorithm and averaging algorithm on MSDCD dataset in **Table S15**.

**Table S1** The statistical information of six independent test datasets.

| Dataset | Residue Level | | Protein Level | |
| --- | --- | --- | --- | --- |
|  | #D(percent)^a^ | #O(percent)^b^ | #LD(percent)^c^ | #SD(percent)^d^ |
| MXD494 | 44087(22.4%) | 152414(77.6%) | 248(50.2%) | 246(49.8%) |
| SL329 | 39544(42.4%) | 51292(57.6%) | 234(71.1%) | 95(28.9%) |
| DISORDER723 | 13526(6.3%) | 201703(93.7%) | 56(7.7%) | 667(92.3%) |
| Disprot504 | 74454(24.7%) | 226992(75.3%) | 504(100%) | 0(0%) |
| CASP | 3929(7.8%) | 46344(92.2%) | 18(8.5%) | 193(91.5%) |
| MSDCD | 175540(20.5%) | 678745(79.5%) | 1060(46.9%) | 1201(53.1%) |
| CAID | 54878(30.8%) | 123246(69.2%) | 437(67.0%) | 215(33%) |

^a^ represents the disordered residue;

^b^ represents the ordered residue;

^c^ represents the LDR proteins;

^d^ represents the SDR proteins.

**Table S2** Performance of various methods on MXD494 independent test dataset

| Predictor | Sn | Sp | BACC | MCC | AUC | Rank | | |
| --- | --- | --- | --- | --- | --- | --- | --- | --- |
|  |  |  |  |  |  | AUC | BACC | MCC |
| DeepIDP-2L(18) | 0.737 | 0.776 | 0.757 | 0.452 | **0.825** | 1 | 2 | 4 |
| IDP-Seq2Seq(13) | 0.743 | 0.791 | **0.767** | **0.475** | **0.825** | 1 | 1 | 1 |
| MFDp(9) | 0.746 | 0.768 | 0.757 | 0.451 | 0.821 | 3 | 2 | 5 |
| MD(16) | 0.673 | 0.813 | 0.743 | 0.444 | 0.821 | 3 | 6 | 6 |
| RFPR-IDP(17) | 0.749 | 0.758 | 0.754 | 0.442 | 0.821 | 3 | 4 | 7 |
| SPOT-Disorder(7) | 0.626 | 0.851 | 0.739 | 0.457 | 0.813 | 6 | 8 | 3 |
| SPINE-D(15) | 0.787 | 0.698 | 0.742 | 0.411 | 0.803 | 7 | 7 | 9 |
| AUCpreD(8) | 0.521 | 0.881 | 0.701 | 0.411 | 0.800 | 8 | 14 | 9 |
| DISOPRED3(6) | 0.622 | 0.820 | 0.721 | 0.410 | 0.800 | 8 | 11 | 11 |
| IDP-FSP(20) | 0.670 | 0.831 | 0.751 | 0.465 | 0.794 | 10 | 5 | 2 |
| PONDR-FIT(21) | 0.631 | 0.821 | 0.726 | 0.419 | 0.790 | 11 | 9 | 8 |
| IUPred-long(12) | 0.581 | 0.841 | 0.711 | 0.405 | 0.784 | 12 | 12 | 13 |
| DISOPRED2(10) | 0.647 | 0.800 | 0.724 | 0.406 | 0.781 | 13 | 10 | 12 |
| IUPred-short(12) | 0.522 | 0.866 | 0.694 | 0.389 | 0.781 | 13 | 15 | 14 |
| DISpro(19) | 0.303 | **0.940** | 0.622 | 0.318 | 0.775 | 15 | 18 | 17 |
| RONN(22) | 0.664 | 0.754 | 0.709 | 0.368 | 0.764 | 16 | 13 | 15 |
| Ucon(23) | 0.554 | 0.787 | 0.671 | 0.313 | 0.741 | 17 | 17 | 18 |
| NORSnet(24) | 0.532 | 0.829 | 0.681 | 0.347 | 0.738 | 18 | 16 | 16 |
| PROFbval(25) | **0.835** | 0.387 | 0.611 | 0.196 | 0.697 | 19 | 19 | 19 |

**Table S3** Performance of various methods on SL329 independent test dataset

| Predictor | Sn | Sp | BACC | MCC | AUC | Rank | | |
| --- | --- | --- | --- | --- | --- | --- | --- | --- |
|  |  |  |  |  |  | AUC | BACC | MCC |
| DeepIDP-2L(18) | 0.73 | 0.93 | **0.828** | **0.68** | **0.904** | 1 | 1 | 1 |
| SPOT-Disorder(7) | 0.65 | **0.96** | 0.805 | 0.65 | 0.901 | 2 | 6 | 3 |
| IDP-Seq2Seq(13) | 0.71 | 0.92 | 0.822 | 0.67 | 0.899 | 3 | 2 | 2 |
| AUCpreD(8) | 0.63 | **0.96** | 0.795 | 0.64 | 0.887 | 4 | 8 | 4 |
| SPINE-D(15) | 0.82 | 0.80 | 0.815 | 0.61 | 0.886 | 5 | 4 | 8 |
| DISOPRED3(6) | 0.67 | 0.92 | 0.796 | 0.62 | 0.880 | 6 | 7 | 6 |
| RFPR-IDP(17) | 0.78 | 0.84 | 0.809 | 0.62 | 0.879 | 7 | 5 | 6 |
| MFDp(9) | **0.88** | 0.62 | 0.750 | 0.51 | 0.873 | 8 | 14 | 13 |
| MD(16) | 0.66 | 0.89 | 0.775 | 0.58 | 0.864 | 9 | 10 | 10 |
| IDP-FSP(20) | 0.75 | 0.89 | 0.821 | 0.65 | 0.864 | 9 | 3 | 5 |
| DISOPRED2(10) | 0.69 | 0.90 | 0.795 | 0.59 | 0.858 | 11 | 8 | 9 |
| DISOClust(11) | 0.81 | 0.70 | 0.755 | 0.51 | 0.846 | 12 | 13 | 13 |
| PONDR-FIT(21) | 0.61 | 0.91 | 0.760 | 0.55 | 0.843 | 13 | 11 | 11 |
| IUpred-long(12) | 0.60 | 0.92 | 0.760 | 0.55 | 0.839 | 14 | 11 | 11 |
| IUpred-short(12) | 0.50 | 0.94 | 0.720 | 0.50 | 0.829 | 15 | 16 | 16 |
| NORSnet(24) | 0.54 | 0.92 | 0.730 | 0.51 | 0.815 | 16 | 15 | 13 |
| Ucon(23) | 0.59 | 0.81 | 0.700 | 0.42 | 0.779 | 17 | 17 | 17 |
| PONDERVL-XT(26) | 0.59 | 0.78 | 0.685 | 0.38 | 0.755 | 18 | 18 | 18 |

**Table S4.** Performance of various methods on DISORDER723 independent test dataset

| Predictor | Sn | Sp | BACC | MCC | AUC | Rank | | |
| --- | --- | --- | --- | --- | --- | --- | --- | --- |
|  |  |  |  |  |  | AUC | BACC | MCC |
| DeepIDP-2L(18) | 0.615 | 0.962 | 0.789 | 0.529 | **0.914** | 1 | 2 | 4 |
| IDP-Seq2Seq(13) | 0.618 | 0.955 | 0.787 | 0.511 | 0.906 | 3 | 3 | 6 |
| AUCpreD(8) | 0.580 | 0.974 | 0.777 | **0.564** | 0.914 | 1 | 4 | 1 |
| DISOPRED3(6) | 0.452 | **0.986** | 0.719 | 0.536 | 0.899 | 4 | 7 | 2 |
| SPOT-Disorder(7) | 0.470 | 0.983 | 0.726 | 0.531 | 0.898 | 5 | 6 | 3 |
| RFPR-IDP(17) | 0.522 | 0.974 | 0.748 | 0.517 | 0.898 | 6 | 5 | 5 |
| SPINE-D(15) | **0.779** | 0.840 | **0.810** | 0.376 | 0.891 | 7 | 1 | 8 |
| IUPred-Short(12) | 0.495 | 0.943 | 0.719 | 0.382 | 0.810 | 8 | 7 | 7 |
| IUPred-Long(12) | 0.298 | 0.949 | 0.623 | 0.247 | 0.721 | 9 | 9 | 9 |

**Table S5.** Performance of various methods on Disprot504 independent test dataset

| Predictor | Sn | Sp | BACC | MCC | AUC | Rank | | |
| --- | --- | --- | --- | --- | --- | --- | --- | --- |
|  |  |  |  |  |  | AUC | BACC | MCC |
| DeepIDP-2L(18) | 0.688 | 0.718 | **0.703** | **0.361** | **0.758** | 1 | 1 | 1 |
| IDP-Seq2Seq(13) | 0.646 | 0.727 | 0.686 | 0.334 | 0.741 | 2 | 2 | 2 |
| SPINE-D(15) | **0.752** | 0.613 | 0.683 | 0.315 | 0.738 | 3 | 3 | 5 |
| SPOT-Disorder(7) | 0.594 | 0.761 | 0.677 | 0.326 | 0.732 | 4 | 5 | 3 |
| AUCpreD(8) | 0.497 | **0.822** | 0.660 | 0.315 | 0.729 | 5 | 7 | 5 |
| IUCpred-Long(12) | 0.575 | 0.772 | 0.674 | 0.323 | 0.725 | 6 | 6 | 4 |
| RFPR-IDP(17) | 0.723 | 0.634 | 0.681 | 0.314 | 0.720 | 7 | 4 | 7 |
| IUCpred-Short(12) | 0.482 | 0.817 | 0.649 | 0.295 | 0.718 | 8 | 8 | 8 |
| DISOPRED3(6) | 0.510 | 0.773 | 0.641 | 0.267 | 0.697 | 9 | 9 | 9 |

**Table S6.** Performance of various methods on CASP independent test dataset

| Predictor | Sn | Sp | BACC | MCC | AUC | Rank | | |
| --- | --- | --- | --- | --- | --- | --- | --- | --- |
|  |  |  |  |  |  | AUC | BACC | MCC |
| DeepIDP-2L(18) | 0.554 | 0.964 | **0.759** | **0.522** | **0.890** | 1 | 1 | 1 |
| SPOT-Disorder(7) | 0.333 | 0.985 | 0.659 | 0.434 | 0.866 | 2 | 7 | 5 |
| IDP-Seq2Seq(13) | 0.469 | 0.965 | 0.717 | 0.459 | 0.863 | 3 | 3 | 4 |
| AUCpreD(8) | 0.428 | 0.978 | 0.703 | 0.483 | 0.863 | 4 | 4 | 2 |
| DISOPRED3(6) | 0.329 | **0.991** | 0.660 | 0.472 | 0.853 | 5 | 6 | 3 |
| RFPR-IDP(17) | 0.400 | 0.963 | 0.682 | 0.395 | 0.845 | 6 | 5 | 6 |
| SPINE-D(15) | **0.652** | 0.840 | 0.746 | 0.331 | 0.826 | 7 | 2 | 7 |
| IUCpred-Short(12) | 0.317 | 0.952 | 0.635 | 0.286 | 0.657 | 8 | 8 | 8 |
| IUCpred-Long(12) | 0.174 | 0.957 | 0.565 | 0.156 | 0.587 | 9 | 9 | 9 |

**Table S7** The statistical information of five validation datasets for optimizing the weights of base methods by multi-objective genetic algorithm

| Datasets | Residue Level | | Protein Level | |
| --- | --- | --- | --- | --- |
|  | #D(percent)^a^ | #O(percent)^b^ | #LD(percent)^c^ | #SD(percent)^d^ |
| $\mathbb{S}_{1}^{Validation}$ | 12504(26.8%) | 34159(73.2%) | 144(100.0%) | 0(0.0%) |
| $\mathbb{S}_{2}^{Validation}$ | 8268(21.1%) | 30869(78.9%) | 96(75.0%) | 48(25.0%) |
| $\mathbb{S}_{3}^{Validation}$ | 7898(21.0%) | 29681(79.0%) | 72(50.0%) | 72(50.0%) |
| $\mathbb{S}_{4}^{Validation}$ | 5407(13.7%) | 33921(86.3%) | 48(25.0%) | 96(75.0%) |
| $\mathbb{S}_{5}^{Validation}$ | 2157(6.3%) | 31820(93.7%) | 0(0.0%) | 144(100.0%) |

^a^ represents the disordered residue;

^b^ represents the ordered residue;

^c^ represents the LDR proteins;

^d^ represents the SDR proteins.

**Table S8** The statistical information of training and validation datasets

| Dataset | Residue Level | | Protein Level | |
| --- | --- | --- | --- | --- |
|  | #D(percent)^a^ | #O(percent)^b^ | #LD(percent)^c^ | #SD(percent)^d^ |
| $\mathbb{S}_{long}^{Train}$ | 100586(26.3%) | 281964(73.7%) | 872(100.0%) | 0(0.0%) |
| $\mathbb{S}_{Short}^{Train}$ | 21463(5.27%) | 385559(94.7%) | 0(0.0%) | 3488(100.0%) |
| $\mathbb{S}_{all}^{Train}$ | 122049(18.3%) | 667523(81.7%) | 872(20%) | 3488(80%) |
| $\mathbb{S}_{long}^{Validation}$ | 12504(26.8%) | 34159(73.2%) | 144(100.0%) | 0(0.0%) |
| $\mathbb{S}_{short}^{Validation}$ | 16578(6.4%) | 242589(93.6%) | 0(0.0%) | 1085(100.0%) |
| $\mathbb{S}_{all}^{Validation}$ | 29082(9.5%) | 276748(90.5%) | 144(11.7%) | 1085(88.3%) |

^a^ represents the disordered residue;

^b^ represents the ordered residue;

^c^ represents the LDR proteins;

^d^ represents the SDR proteins.

**Table S9.** The statistical information of eight test datasets simulating real-world application scenarios**.**

| Percentage^a^ | #D^b^ | #O^c^ |
| --- | --- | --- |
|  |  |  |
| 20% | 83 | 329 |
| 25% | 110 | 329 |
| 30% | 141 | 329 |
| 35% | 177 | 329 |
| 40% | 219 | 329 |
| 45% | 269 | 329 |
| 50% | 329 | 329 |
| 55% | 402 | 329 |

^a^ represents the percentage of disordered proteins in the corresponding dataset

^b^ represents the disordered proteins;

^c^ represents the fully ordered proteins;

**Table S10.** Performance of different HAN predictors based on different combing features on the validation dataset $\mathbb{S}_{long}^{Validation}$ .

| Different combing features | AUC |
| --- | --- |
| PSSM | 0.845 |
| PSSM+PSFM | 0.850 |
| PSSM+PSFM+HHM | 0.865 |
| PSSM+PSFM+HHM+SS | 0.868 |
| PSSM+PSFM+HHM+SS+SEVEN | 0.870 |
| PSSM+PSFM+HHM+SS+SEVEN+SA | 0.871 |
| PSSM+PSFM+HHM+SS+SEVEN+SA+CCM | 0.875 |

**Table S11.** Performance of different CAN predictors based on different combing features on the validation dataset $\mathbb{S}_{short}^{Validation}$.

| Different combing features | AUC |
| --- | --- |
| PSSM | 0.886 |
| PSSM+PSFM | 0.889 |
| PSSM+PSFM+HHM | 0.901 |
| PSSM+PSFM+HHM+SS+CN+HSE | 0.910 |
| PSSM+PSFM+HHM+ SS+CN+HSE+SEVEN | 0.915 |
| PSSM+PSFM+HHM+ SS+CN+HSE+SEVEN+SA | 0.918 |
| PSSM+PSFM+HHM+ SS+CN+HSE+SEVEN+SA+CCM | 0.923 |

**Table S12.** Performance of different IDP-Seq2Seq predictors based on different combing features on the validation dataset $\mathbb{S}_{all}^{Validation}$.

| Different combing features | AUC |
| --- | --- |
| PSSM | 0.875 |
| PSSM+PSFM | 0.877 |
| PSSM+PSFM+HHM | 0.885 |
| PSSM+PSFM+HHM+SS+CN+HSE | 0.889 |
| PSSM+PSFM+HHM+ SS+CN+HSE+SEVEN | 0.891 |
| PSSM+PSFM+HHM+ SS+CN+HSE+SEVEN+SA | 0.894 |
| PSSM+PSFM+HHM+ SS+CN+HSE+SEVEN+SA+CCM | 0.896 |

**Table S13.** Performance of different CNN-LSTM predictors based on different combing features on the validation dataset $\mathbb{S}_{all}^{Validation}$.

| Different combing features | AUC |
| --- | --- |
| PSSM | 0.872 |
| PSSM+PSFM | 0.875 |
| PSSM+PSFM+HHM | 0.879 |
| PSSM+PSFM+HHM+SS+CN+HSE | 0.881 |
| PSSM+PSFM+HHM+ SS+CN+HSE+SEVEN | 0.883 |
| PSSM+PSFM+HHM+ SS+CN+HSE+SEVEN+SA | 0.884 |
| PSSM+PSFM+HHM+ SS+CN+HSE+SEVEN+SA+CCM | 0.890 |

**Table S14.** Performance of different LSTM-CNN predictors based on different combing features on the validation dataset $\mathbb{S}_{all}^{Validation}$.

| Different combing features | AUC |
| --- | --- |
| PSSM | 0.874 |
| PSSM+PSFM | 0.876 |
| PSSM+PSFM+HHM | 0.884 |
| PSSM+PSFM+HHM+SS+CN+HSE | 0.885 |
| PSSM+PSFM+HHM+ SS+CN+HSE+SEVEN | 0.890 |
| PSSM+PSFM+HHM+ SS+CN+HSE+SEVEN+SA | 0.892 |
| PSSM+PSFM+HHM+ SS+CN+HSE+SEVEN+SA+CCM | 0.893 |

**Table S15**. The performance of multi-objective genetic algorithm and averaging algorithm on MSDCD dataset

| Model | Sn | Sp | BACC | MCC | AUC |
| --- | --- | --- | --- | --- | --- |
| IDP-Fusion^a^ | 0.685 | 0.851 | 0.768 | 0.494 | 0.846 |
| IDP-Fusion^b^ | 0.704 | 0.822 | 0.764 | 0.472 | 0.837 |

^a^ IDP-Fusion model with average ensemble strategy;

^b^ IDP-Fusion model with multi-objective genetic algorithm;
